# The Management of Intraabdominal Testis: A Survey of the World Federation of Associations of Pediatric Surgeons (WOFAPS) Practices

**DOI:** 10.3389/fped.2022.928069

**Published:** 2022-06-29

**Authors:** Sameh Shehata, Faruk Hadziselimovic, Doaa Khater, Mostafa Kotb

**Affiliations:** ^1^Department of Pediatric Surgery, Alexandria Faculty of Medicine, Alexandria, Egypt; ^2^Department of Pediatrics, Children's Day Care Center Liestal, Cryptorchidism Research Institute, Liestal, Switzerland; ^3^Department of Pediatric Endocrinology, Alexandria Faculty of Medicine, Alexandria, Egypt

**Keywords:** intraabdominal testis, RedCap, WOFAPS, survey, practices

## Abstract

**Background and Objective:**

The optimal treatment protocol of intraabdominal testis is still a matter of debate and until now there are a lot of areas of controversy as regards this challenging subtype. The aim of this report is to document current practice patterns among surgeons from different continents through an online Redcap survey supervised the World Federation of the Association of Pediatric Surgeons (WOFAPS).

**Methods:**

A 16-question-survey related to the management of intraabdominal testis was created and administered *via* RedCap. The WOFAPS headquarters sent an email to all members inviting voluntary survey participation. Data were entered using Microsoft EXCEL spreadsheet and analyzed. Descriptive statistics were performed for each survey item.

**Results:**

There were 436 WOFAPS members who participated in this study with a response rate of 29%, and the vast majority were pediatric surgeons. Only 13% tried to use hormone therapy aiming to induce testicular descent or to improve future fertility. The choices of various surgical techniques were noted. During laparoscopy, if vessels and cord structure were seen entering the ipsilateral internal inguinal ring, most respondents chose to explore the groin. On the other hand, should there was an absent or atrophic testis, the respondents were split on whether to perform a contralateral orchiopexy.

**Conclusion:**

This survey describes the current practices of a sample of pediatric surgeons and urologists in the management of intraabdominal testis. The use of hormonal treatment, timing of fixation and management in case of passing through vas and vessels through DIR were undisputable. However, management of low-lying and peeing testis together with the management of contralateral testis were still debatable.

## Introduction

Cryptorchidism or undescended testis is the most common genitourinary anomaly affecting boys with an overall incidence of around 3% in full term neonates, reaching up to 30% in pre-mature boys (1). During the first months of life, spontaneous normal descent process of the testes may continue. Cryptorchidism is detected in 1–2% by 3-month of age, and drops to 0.8–1.2% in boys at the age of 1 year (2). Approximately 20% of the testicular maldescent is not palpable (3). A non-palpable testis could be either vanishing testis (due to intra-uterine demise), true monorchia (due to agenesis), intra-abdominal testis, or inguinal testis showing a different grade of dysplasia, atrophy or dysplasia (4).

While the management of boys with palpable undescended testis is more or less standardized, the optimal treatment protocol of intraabdominal testis is still a matter of debate and until now there are a lot of areas of controversy as regards this challenging subtype (5). Among these areas are the role of hormonal therapy, the optimal surgical procedures should the testis was viable using laparoscopy and the ideal management scenario in cases of passing through and absent testis. The aim of this report is to document current practice patterns among pediatric surgeons and urologists from different continents through an online redcap survey supervised the World Federation of the Association of Pediatric Surgeons (WOFAPS).

## Methods

A 16-question-survey regarding respondent demographics and practices related to the management of intraabdominal testis was created and administered *via* RedCap, a web application for building and managing online surveys and databases. The first four demographic questions were required, but all other questions were answered at participant discretion. The survey was developed and screened through the WOFAPS Outcomes and Evidence Based Practice Committee prior to dissemination. The headquarters of WOFAPS sent an email on 4th of April 2021 to all WOFAPS members inviting voluntary survey participation. Collective survey answers were tabulated and reported as frequencies and percentages. Data were entered using Microsoft EXCEL spreadsheet and analyzed using IBM SPSS software package version 20.0. (Armonk, NY: IBM Corp). Descriptive statistics were performed for each survey item.

## Results

### Respondent Demographics

A total of 436 of 1,503 WOFAPS members (29%) who received the survey responded with the majority being from Latin America and Asia (both contribute to around one-third). This was followed by Africa and Europe with minimal contribution by North America and Australia. Around 92% were pediatric surgeon, 7% pediatric urologist, and 1% general surgeons. Approximately two-fifth of the respondents had 21–30 years of experience.

The results of the response to the survey is summarized in Table 1. The highlights of the results revealed some important agreements as well as discrepancies. Regarding the use of hormonal treatment, only 13% tried to use hormone therapy to induce passage of intra-abdominal testicles or to try to improve future fertility. Most respondents would perform surgery after age 6 months. The choices of various surgical techniques were noted including Fowler-Stephen's, Shehata's technique and lap assisted single stage vessel sparing orchiopexy. In cases where there is no palpable testicle and laparoscopy shows vessels and cord structure on laparoscopy entering the ipsilateral internal inguinal ring, most respondents chose to explore the groin. On the other hand, if there was an absent or atrophic testis, the respondents were almost split on whether to perform a contralateral orchiopexy (54% against, 46% for).

**Table 1:** Management practices evaluated in the survey.

**Question**	**Number (%)**
**Do you use hormonal treatment to induce testicular descent?**	
Yes	57 (13.1)
No	379 (86.9)
**Do you use hormonal treatment to improve future fertility?**	
Yes	31 (7.1)
No	405 (92.9)
**What is the preferred timing for operation?**	
6–9 months	183 (41.9)
10–12 months	170 (39.1)
12–18 months	78 (17.9)
>18 months	5 (1.1)
**Which operation do you usually perform?**	
Fowler-Stephens (FS) only	292 (67.0)
Shehata technique only	86 (19.7)
Both	58 (13.3)
**What is the waiting period between stages in FS orchiopexy?**	
3 months	39 (11.1)
3–6 months	205 (58.6)
More than 6 months	106 (24.3)
**What is the waiting period between stages in Shehata's orchiopexy?**	
2 weeks	2 (1.4)
2–4 weeks	14 (9.7)
4–8 weeks	51 (35.4)
8–12 weeks	77 (53.5)
**What is the preferred technique for testis within 3 cm from DIR?**	
Two-stage FS	157 (36.0)
Lap assisted single stage vessel sparing orchiopexy	153 (35.1)
Shehata technique	80 (18.4)
One-stage FS	45 (10.3)
Microvascular autotransplantation	1 (0.2)
**What is the preferred technique for peeping testis?**	
Inguinal orchiopexy	220 (50.4)
Lap assisted single stage orchiopexy	176 (40.4)
Lap assisted FS	24 (5.3)
Lap assisted Shehata technique	16 (3.9)
**What to do if you find a vas and vessels passing through closed DIR?**	
Explore the inguinal canal	328 (75.2)
Do not explore the inguinal canal	108 (24.8)
**What to do if you find a vas and vessels passing through open DIR (patent processus vaginalis)?**	
Explore the inguinal canal	400 (91.7)
Do not explore the inguinal canal	36 (8.3)
**Would you perform prophylactic contralateral pexy in absent testis?**	
Yes	200 (45.9)
No	236 (54.1)
**Have you ever seen torsion in a single testis?**	
Yes	99 (22.7)
No	337 (77.3)

## Discussion

This survey describes the current practices of a sample of pediatric surgeons and urologists in the management of intraabdominal testis. Points of interest were the use of hormonal therapy, timing of surgery, and surgical options in different scenarios during diagnostic laparoscopy.

### Hormonal Treatment

The majority of the respondents chose not to use hormonal therapy by any means during management of an intraabdominal testis, where only 13% use hormonal therapy to induce testicular descent and 7% to improve future fertility. The hormonal treatment of cryptorchidism is based on the hormonal reliance of testicular descent. Human chorionic gonadotropin (hCG), gonadotropin releasing hormone (GnRH, luteinizing hormone releasing hormone—LHRH) or a combination of both are the most used hormonal therapies (6).

The main reason for not recommending hormonal treatment of the undescended testis is the low success rate of this treatment (up to 20%), (7) which after follow-up, drops to 15% as a result of secondary re-ascent of the testis (8). Nevertheless, this was not compatible by the randomized study performed by Höcht et al. (9) who reported an initial descend of 59% (17/29), which dropped slightly after 10 years to 52% after initial treatment using LH-RH nasal sprays. In the often cited study performed by Cortes et al. (10) the use of hCG in cryptorchidism was claimed to be acco with higher apoptosis rate of spermatogonia and a 50% smaller testicular volume compared to cryptorchid testes not treated with hCG. Therefore, based on the aforementioned causes, combined with the possible deleterious side effects and the lack of published long-term outcome data, recent AUA guidelines along with the Nordic consensus panel defer the use of hormonal therapy to facilitate testicular descent (7, 11). Likewise, the European Association of Urology (EAU)/European Society for Pediatric Urology (ESPU) Guidelines Committee stated that there is no consensus on utilizing hormonal treatment for testicular descent (Level 2 evidence, Grade B recommendation) (12).

On the other hand, proponents of the role of hormonal therapy demonstrated that cryptorchidism is a serious andrological problem even if it is a unilateral one. This is supported by the fact that even after successful surgery, azoospermia is 25 times more common in unilateral and 80 times more common in bilateral cryptorchidism in comparison with the control population (13). Thus, it is recommended that hormonal therapy should be the first therapeutic choice because it may avoid resorting to surgery, as claimed by Höcht et al. with the highest success reported when the testis was in the pre-scrotal position (9, 14). Moreover, on comparing those who received LH-RH spray after successful orchiopexy with those who underwent surgery alone, 86% of the former group was within the normal range, whilst all the latter group remained oligospermic (15). Hence, a post-surgical hormonal treatment for the high infertility and azoospermia patients who underwent successful early orchiopexy has been recommended (16).

### Timing of Surgery

The optimal timing for intervention was not a subject of controversy in the survey as 82% reported to perform orchiopexy from 6 to 12 months (42% from 6 to 9 months, 39% from 9 to 12 months), whilst only one-fifth prefer at 12–18 months.

Because of the high incidence of spontaneous descent during the first few months of life, surgery for undescended testes diagnosed at birth should not be performed before 6 months of age (Level 2 evidence, Grade B recommendation). Wenzler et al. (17) performed a 10-year, retrospective study of 1,235 boys with cryptorchidism and they found that should the testis was still undescended by 2 months of age, the chance of spontaneous descent dropped drastically, with the chance of descent after 6 months of age is almost nil. Hence, if the testis remains undescended by the age of 6 months, orchidopexy should be performed as early as 12 months, (17) and up to a maximal cutoff of 18 months of age as recommended by the 2014 AUA update on cryptorchidism (11). This is because it appears that the longer cryptorchid boys wait to have orchidopexy, the higher the risk of having a paucity of germ cells in their testes, (18) elevating the risk of infertility from 79 to almost 100% (19). In addition, Kollin et al. (20) showed an improvement of testicular growth with early orchidopexy (9 months) when compared to orchidopexy at the age of 3 years. Another factor is that the distance required to lower the testicles in older boys becomes too long, which increases the risk and difficulty of surgery (21).

### Surgical Approach

#### Testis Found Within 3 cm From Deep Inguinal Ring (DIR)

This distance is measured from the lowest point in which the testis can reach the DIR without tension using a piece of suture material and then measured externally on a scale. Two techniques, the two-stage Fowler-Stephens (FS) and the lap assisted single stage vessel sparing orchiopexy, were almost equally preferred by around 70% of the respondents in management of low-lying testis. These are followed by Shehata technique in 18% and one-stage FS in a tenth of the surgeons.

For the low-lying abdominal testis (<3 cm from DIR), one-stage orchiopexy is a suitable option. The key steps are mobilization of the peritoneum, dividing the gubernaculum and redirecting the spermatic cord medial to the inguinal canal (Prentiss maneuver). Excellent results are reported in low-lying abdominal testis, for instance, Baker et al. reported success in 173/175 cases (97.2%) and Radmayr et al. in 28/28 cases (100%) (22, 23).

In 1959, Fowler and Stephens (24) reported the single-stage testicular descent fixation following spermatic vascular ligation based on the fact that vas deferens generally is sufficiently long to allow the IAT to reach the scrotum. Later, modifications of the technique was done, where the testes are kept in the abdomen *in situ* without any treatment after ligating the spermatic vessels, followed by staged surgery. As regards the outcome, Esposito et al. (25) found that 10/12 (83%) had a viable testis in the scrotum. However, despite the good vascularization detected on color doppler ultrasound, the operated testis was always smaller in size than the normal testis. Additionally, Rosito et al. (26) found that the number of spermatogonia and seminiferous tubules was reduced significantly 6 months after ligation of the spermatic vessels. Despite these reported drawbacks, FS technique is still commonly used worldwide.

Whether one- or two- stage FSO is superior is not yet fully determined. In a meta-analysis performed by Elyas et al. and by Wayne et al. (27, 28) two-stage FS was minimally but significantly superior; hence, the choice between one- or two- stage FSO repair should be according to the surgeon's preference. Opposite findings were found by Elzeneini et al. (29) who found that 25% of the testes were atrophic and 50% only were scrotal after one-stage FS compared to 12% atrophic and 96% in scrotal position after one-stage FS.

On the other hand, Shehata technique entails laparoscopic staged traction procedure without the need for vessel ligation. In this operation, a suture fixation of the testis to the anterior abdominal wall, medial to the contralateral anterior superior iliac spine with 2nd stage laparoscopic orchiopexy performed around 12-weeks later. This fixation is thought to use the gravity of the intestine to continuously and gently produce traction to the testicular vessels. The overall success rate was 84 percent in 140 testes as reported by the authors, with younger children and those with lower testicles having higher success rate (30). In another study performed by Abouheba et al. (31) neither testicular atrophy by undue tension (due to preserved gonadal vessels) nor internal herniation complicated the traction period or follow-up periods.

Fortunately, post-operative complications are relatively uncommon, where testicular atrophy being the most serious. Typically, it results from an iatrogenic injury to the spermatic vessels, tension on the cord, with resultant ischemia, iatrogenic torsion, and clipping of the gonadal vessels as an essential step of FSO. Other less common complications include iatrogenic testicular ascent and vas deferens injury (12).

#### Peeping Testis

Peeping testis represents a diagnostic challenge and a therapeutic dilemma. Open surgical treatment of such testes is the most popular approach among pediatric urologists and surgeons. Around half of the respondents of this survey still chose the inguinal approach for management of peeping testis. Nevertheless, due to the difficult surgical mobilization of some peeping testes along with significant complications such as testicular retraction/atrophy (3–18%), some surgeons believe that lap-assisted orchiopexy for such high inguinal testes is an attractive alternative approach. The most preferred lap-assisted technique in our survey was the lap assisted one stage orchiopexy (40% of the respondents), followed by lap assisted FS and lap assisted Shehata techniques.

The potential advantages of laparoscopy in such situations are a better magnification, and a wide range of testicular dissection with facilitation of the Prentiss maneuver to allow satisfactory orchiopexy without significant vascular compromise. In a study performed by Elderwy et al. (32) open and laparoscopic orchiopexy procedures for peeping testes were fairly comparable; though laparoscopy was relatively more effective, as two re-do orchiopexies were required in the open surgical group.

#### Passing Through Vas and Vessels Through a Closed or Open DIR

When cord structures found entering the DIR, some surgeons prefer not to explore the inguinal canal regardless the patency of the DIR claiming that even there was a testicular nubbin, excision is not necessary as it lacks viable germ cells (33). Nonetheless, the majority prefers to perform an inguinal exploration in this case to search for an ectopic or an intracanalicular atrophic testis, where the latter can be a risk factor for developing testicular cancer (32). An inguinal exploration is recommended in obese patients as well, because these patients might have a small normal testis in the inguinal canal (34, 35).

Esposito et al. (36) prefer laparoscopic inguinal exploration if the DIR is open, as the intraabdominal testis might have been migrated into the inguinal canal by the effect of abdominal insufflation, leaving exploration *via* the open surgery to patients with a closed DIR. In a more recent study, laparoscopic inguinal exploration was feasible in cases of impalpable UDTs, irrespective of the status of the DIR or the appearance of the testicular vessels; however, this study was limited by the small number of included cases (37).

#### Management of Contralateral Testis in Case of a Solitary Testis

The management of contralateral testis in case of a solitary testis continued to be a debatable issue and perhaps this is the most hotly disputed one in our survey. Surgeons who do not prefer to fix the contralateral testis are slightly higher than those who prefer contralateral orchiopexy (54% vs. 46%). The rationale of fixation is to prevent the disastrous event of torsion of the solitary testis, where, in our survey, around quarter of the respondents reported seeing torsion of a single testis. On the other hand, those who argue that contralateral fixation is not necessary claim that prevalence of contralateral bell clapper anomaly is exceedingly small, substantiating an insufficient risk of subsequent torsion to justify routine fixation of the solitary testis. In a research conducted by Martin and Rushton (38), 50 children with a testicular nubbin underwent contralateral scrotal exploration to assess the risk of future torsion, and only one patient had a partial bell clapper abnormality in the contralateral solitary testis. Thus, this issue should be discussed with the patient parents or legal guardian prior to intervention (Level 5 evidence, Grade D recommendation) (39).

To sum up, this survey describes the current practices of a sample of pediatric surgeons and urologists in the management of intraabdominal testis. The use of hormonal treatment, timing of fixation and management in case of passing through vas and vessels through DIR were generally agreeable. However, management of low-lying and peeing testis together with the management of contralateral testis were still areas of debate in this survey.

## Data Availability Statement

The raw data supporting the conclusions of this article will be made available by the authors, without undue reservation.

## Author Contributions

SS: protocol development and critical revision. FH: critical revision. DK and MK: data collection and manuscript writing. All authors contributed to the article and approved the submitted version.

## Conflict of Interest

The authors declare that the research was conducted in the absence of any commercial or financial relationships that could be construed as a potential conflict of interest.

## Publisher's Note

All claims expressed in this article are solely those of the authors and do not necessarily represent those of their affiliated organizations, or those of the publisher, the editors and the reviewers. Any product that may be evaluated in this article, or claim that may be made by its manufacturer, is not guaranteed or endorsed by the publisher.

## References

[1] Sijstermans K Hack WWM Meijer RW Sijstermans K Hack WW Meijer RW. The frequency of undescended testis from birth to adulthood: a review. Int J Androl. (2008) 31:1–11. 10.1111/j.1365-2605.2007.00770.x 17488243

[2] Virtanen HE Bjerknes R Cortes D Jørgensen N Rajpert-De Meyts E Thorsson AV. Cryptorchidism: classification, prevalence and long-term consequences. Acta Paediatr. (2007) 96:611–6. 10.1111/j.1651-2227.2007.00241.x 17462053

[3] Niedzielski JK Oszukowska E Słowikowska-Hilczer J. Undescended testis–current trends and guidelines: a review of the literature. Arch Med Sci. (2016) 12:667–77. 10.5114/aoms.2016.59940 27279862 PMC4889701

[4] Sepúlveda X Egaña PJ. Current management of non-palpable testes: a literature review and clinical results. Transl Pediatr. (2016) 5:233–9. 10.21037/tp.2016.10.06 27867845 PMC5107370

[5] Elder JS. Surgical management of the undescended testis: recent advances and controversies. Eur J Pediatr Surg. (2016) 26:418–26. 10.1055/s-0036-1592197 27631723

[6] Hadziselimovic F Herzog B. The importance of both an early orchidopexy and germ cell maturation for fertility. Lancet. (2001) 358:1156–7. 10.1016/S0140-6736(01)06274-2 11597673

[7] Ritzén EM Bergh A Bjerknes R Christiansen P Cortes D Haugen SE. Nordic consensus on treatment of undescended testes. Acta Paediatr. (2007) 96:638–43. 10.1111/j.1651-2227.2006.00159.x 17326760

[8] Ong C Hasthorpe S Hutson JM. Germ cell development in the descended and cryptorchid testis and the effects of hormonal manipulation. Pediatr Surg Int. (2005) 21:240–54. 10.1007/s00383-005-1382-0 15726388

[9] Höcht B. LH-RH treatment for cryptorchidism. Randomized study and 10-year follow-up results. Eur J Pediatr. (1987) 146 Suppl 2:S44–6. 10.1007/BF00452871 2891522

[10] Cortes D Thorup J Visfeldt J. Hormonal treatment may harm the germ cells in 1–3-year-old boys with cryptorchidism. J Urol. (2000) 163:1290–2. 10.1016/S0022-5347(05)67763-4 10737531

[11] Kolon TF Herndon CDA Baker LA Baskin LS Baxter CG Cheng EY. Evaluation and treatment of cryptorchidism: AUA guideline. J Urol. (2014) 192:337–45. 10.1016/j.juro.2014.05.005 24857650

[12] Radmayr C Dogan HS Hoebeke P Kocvara R Nijman R Stein R. Management of undescended testes: European association of urology/European society for paediatric urology guidelines. J Pediatr Urol. (2016) 12:335–43. 10.1016/j.jpurol.2016.07.014 27687532

[13] Hadziselimovic F Hoecht B. Testicular histology related to fertility outcome and post-pubertal hormone status in cryptorchidism. Klin Padiatr. (2008) 220:302–7. 10.1055/s-2007-993194 18401814

[14] Penson D Krishnaswami S Jules A McPheeters ML. Effectiveness of hormonal and surgical therapies for cryptorchidism: a systematic review. Pediatrics. (2013) 131:e1897–907. 10.1542/peds.2013-0072 23690511 PMC4074661

[15] Hadziselimovic F. Successful treatment of unilateral cryptorchid boys risking infertility with LH-RH analogue. Int Braz J Urol. (2008) 34:319–28. 10.1590/S1677-55382008000300009 18601762

[16] Hadziselimovic F. Is hormonal treatment of congenital undescended testes justified? A debate. Sex Dev. (2019) 13:3–10. 10.1159/000496418 30721907

[17] Wenzler DL Bloom DA Park JM. What is the rate of spontaneous testicular descent in infants with cryptorchidism? J Urol. (2004) 171:849–51. 10.1097/01.ju.0000106100.21225.d7 14713841

[18] Lee PA Coughlin MT Bellinger MF. Paternity and hormone levels after unilateral cryptorchidism: association with pre-treatment testicular location. J Urol. (2000) 164:1697–701. 10.1016/S0022-5347(05)67087-5 11025752

[19] Cortes D Thorup JM Visfeldt J. Cryptorchidism: aspects of fertility and neoplasms. Horm Res. (2001) 55:21–7. 10.1159/000049959 11423738

[20] Kollin C Hesser U Martin Ritzén E Karpe B. Testicular growth from birth to 2 years of age, and the effect of orchidopexy at age 9 months: a randomized, controlled study. Acta Paediatr. (2006) 95:318–24. 10.1080/08035250500423812 16497643

[21] Liu J Tang R Wang X Sui B Jin Z Xu X. Comparison of two types of staged laparoscopic orchiopexy for high intra-abdominal testes in children: a retrospective study from a single center. Front Pediatr. (2021) 9:677955. 10.3389/fped.2021.677955 34222147 PMC8247650

[22] Baker LA Docimo SG Surer I Peters C Cisek L Diamond DA. A multi-institutional analysis of laparoscopic orchidopexy. BJU Int. (2001) 87:484–9. 10.1046/j.1464-410X.2001.00127.x 11298039

[23] Radmayr C Oswald J Schwentner C Neururer R Peschel R Bartsch G. Long-term outcome of laparoscopically managed non-palpable testes. J Urol. (2003) 170:2409–11. 10.1097/01.ju.0000090024.02762.3d 14634439

[24] Fowler R Stephens FD. The role of testicular vascular anatomy in the salvage of high undescended testes. Aust N Z J Surg. (1959) 29:92–106. 10.1111/j.1445-2197.1959.tb03826.x 13849840

[25] Esposito C Vallone G Savanelli A Settimi A. Long-term outcome of laparoscopic Fowler-Stephens orchiopexy in boys with intra-abdominal testis. J Urol. (2009) 181:1851–6. 10.1016/j.juro.2008.12.003 19233407

[26] Rosito NC Koff WJ da Silva Oliveira TL Cerski CT Salle JL. Volumetric and histological findings in intra-abdominal testes before and after division of spermatic vessels. J Urol. (2004) 171:2430–3. 10.1097/01.ju.0000125242.43762.be 15126869

[27] Elyas R Guerra LA Pike J DeCarli C Betolli M Bass J. Is staging beneficial for Fowler-Stephens orchiopexy? A systematic review. J Urol. (2010) 183:2012–8. 10.1016/j.juro.2010.01.035 20303527

[28] Wayne C Chan E Nasr A. What is the ideal surgical approach for intra-abdominal testes? A systematic review. Pediatr Surg Int. (2015) 31:327–38. 10.1007/s00383-015-3676-1 25663531

[29] Elzeneini WM Mostafa MS Dahab MM Youssef AA AbouZeid AA. How far can one-stage laparoscopic fowler-stephens orchiopexy be implemented in intra-abdominal testes with short spermatic vessels? J Pediatr Urol. (2020) 16:197.e1–7. 10.1016/j.jpurol.2020.01.003 32085874

[30] Shehata S Shalaby R Ismail M Abouheba M Elrouby A. Staged laparoscopic traction-orchiopexy for intraabdominal testis (Shehata technique): stretching the limits for preservation of testicular vasculature. J Pediatr Surg. (2016) 51:211–5. 10.1016/j.jpedsurg.2015.10.063 26655212

[31] Abouheba MA Younis W Elsokary A Roshdy W Waheeb S. Early clinical outcome of staged laparoscopic traction orchidopexy for abdominal testes. J Laparoendosc Adv Surg Tech. (2019) 29:531–7. 10.1089/lap.2018.0171 30807243

[32] Elderwy AA Kurkar A Abdel-Kader MS Abolyosr A Al-Hazmi H Neel KF. Laparoscopic vs. open orchiopexy in the management of peeping testis: a multi-institutional prospective randomized study. J Pediatr Urol. (2014) 10:605–9. 10.1016/j.jpurol.2014.06.006 25042877

[33] Woodford E Eliezer D Deshpande A Kumar R. Is excision of testicular nubbin necessary in vanishing testis syndrome? J Pediatr Surg. (2018) 53:2495–7. 10.1016/j.jpedsurg.2018.08.011 30503248

[34] Taran I Elder JS. Results of orchiopexy for the undescended testis. World J Urol. (2006) 24:231–9. 10.1007/s00345-006-0056-4 16676187

[35] Tackett LD Wacksman J Billmire D Sheldon CA Minevich E. The high intra-abdominal testis: technique and long-term success of laparoscopic testicular autotransplantation. J Endourol. (2002) 16:359–61. 10.1089/089277902760261383 12227909

[36] Esposito C Caldamone AA Settimi A El-Ghoneimi A. Management of boys with non-palpable undescended testis. Nat Clin Pract Urol. (2008) 5:252–60. 10.1038/ncpuro1102 18414455

[37] Pathak M Suchiang B Saxena R Sinha A Rathod K Jadhav A. Laparoscopic inguinal exploration for impalpable undescended testis: can we avoid the open inguinal exploration altogether? J Pediatr Endosc Surg. (2019) 1:167–70. 10.1007/s42804-019-00031-y

[38] Martin A Rushton H. The prevalence of bell clapper anomaly in the solitary testis in cases of prior perinatal torsion. J Urol. (2014) 191:1573–7. 10.1016/j.juro.2013.09.013 24679875

[39] Braga LH Lorenzo AJ Romao RL. Canadian urological association-Pediatric urologists of Canada (CUA-PUC) guideline for the diagnosis, management, and follow-up of cryptorchidism. Can Urol Assoc J. (2017) 11:E251–60. 10.5489/cuaj.4585 28761584 PMC5519382

